# Willingness of Patients With Mental Disorders to Engage in Online Psychotherapy: Multicenter Cross-Sectional Survey

**DOI:** 10.2196/83299

**Published:** 2026-04-07

**Authors:** Lijun Liu, Bingling Gao, Qianqian Li, Huipeng Ren, Miao Pan, Pingfang Zhong, Shixing Li, Chenhong Zhang, Xiaoxi Zhang, Jinmin Liao

**Affiliations:** 1 Department of Psychological Medicine Zhongshan Hospital (Xiamen), Fudan University Xiamen China; 2 Peking University Sixth Hospital, Peking University Institute of Mental Health, NHC Key Laboratory of Mental Health (Peking University), National Clinical Research Center for Mental Disorders (Peking University Sixth Hospital) Beijing China; 3 Department of Psychiatry First Hospital of Hebei Medical University Shijiazhuang, Hebei China; 4 The Second Affiliated Hospital of Xinxiang Medical University Xinxiang, Henan China; 5 The Third People's Hospital of Lincang Lincang, Yunnan China; 6 Ji’ao Brain Hospital of Siping Siping, Jilin China; 7 Shaanxi Nuclear Industry 215 Hospital Xianyang, Shaanxi China; 8 Department of Mental Health Changzhi Medical College Changzhi, Shanxi China

**Keywords:** mental disorders, online psychotherapy, willingness, attitude, stigma

## Abstract

**Background:**

China faces a high prevalence of mental disorders but low treatment uptake, a gap driven by limited awareness and unevenly distributed mental health resources. While online psychotherapy has the potential to expand access, patient willingness remains insufficiently explored.

**Objective:**

This study aimed to investigate the willingness of Chinese patients with mental disorders to engage in online psychotherapy and to identify associated factors.

**Methods:**

A multicenter, cross-sectional survey was conducted using a structured questionnaire to assess the attitudes and willingness of patients with mental disorders in China to engage in online psychotherapy. Willingness to engage in online psychotherapy was assessed using a 0 to 100 rating scale, with higher scores indicating greater willingness. Univariate analysis, correlation analysis, and multivariate linear regression analyses were used to identify factors influencing willingness.

**Results:**

Among 361 eligible participants, the mean willingness score for online psychotherapy was 70 (SD 28.56). In total, 86.4% (n=312) of participants preferred short-term therapy (1 to 10 sessions), while 92.5% (n=334) expected the cost per session to remain less than CNY ¥400 (US $55.50). Participants most preferred therapist-guided online individual therapy (n=142, 39.3%). Convenience (124/361, 34.3%) and perceived anonymity (“no one will know about the illness”; 119/361, 33.0%) were the 2 most commonly reported perceived benefits of online psychotherapy. The leading barrier was concerns about data security and privacy (108/303, 35.6%), followed by difficulty in establishing therapeutic rapport (60/303, 19.8%). The regression analysis revealed that age, self-stigma, satisfaction with current psychiatric medications, and satisfaction with previous online psychotherapy significantly influenced patients’ willingness to seek online psychotherapy.

**Conclusions:**

This multicenter study reveals a high level of willingness to engage in online psychotherapy among Chinese patients, with self-stigma as a key barrier. These findings support the development of tailored services, stigma reduction interventions, and infrastructure investment to enhance mental health care delivery.

## Introduction

Ranked as the second leading cause of disease burden worldwide, mental disorders present a major challenge to global health [1]. However, progress toward universal health coverage is significantly impeded by disparities in mental health services [2]. In China, although the lifetime prevalence of mental disorders among adults is 16.6% [3], the service use rates of mental health services are very low. For example, among individuals with depression, merely 9.5% seek professional help and only 0.5% receive adequate treatment. [4]. This treatment gap is often attributed to factors such as low mental health awareness, social stigma, inequitable resource distribution, and barriers to in-person care [2,4,5].

Driven by digital advances and the COVID-19 pandemic, online mental health services have become mainstream, with a 71% acceptance rate among patients [6]. Online psychotherapy, or telepsychology, includes interventions delivered via video calls, SMS text messaging, and apps, using methods such as cognitive behavioral therapy, dialectical behavior therapy, and mindfulness-based interventions [7,8]. Evidence indicates that online psychotherapy is as effective as in-person therapy for depression, anxiety disorders, posttraumatic stress disorder, and obsessive-compulsive disorder [9]. However, adoption faces barriers such as technical issues, privacy concerns, and challenges in establishing a therapeutic alliance [10-12].

Online psychotherapy presents a valuable opportunity to expand mental health service coverage. However, its implementation in China must consider local cultural contexts and patient-specific needs. Previous studies have focused on single cities, with little data from nationwide, multicenter surveys [13,14]. Previous research has found that age and sex may influence willingness to undergo psychotherapy, while the level of self-stigma is negatively correlated with the intention to seek psychological help [4,15,16]. Further studies have revealed that self-stigma may interfere with the formation of the therapeutic alliance during treatment [17]. Previous experiences with psychotherapy, whether online or in person, may also be significant factors influencing future willingness to engage in online psychological treatment.

In this study, we aimed to conduct a multicenter cross-sectional survey to assess the willingness to engage in online psychotherapy among Chinese patients with mental disorders and to identify the key factors associated with their willingness to use such services. We hypothesize that higher levels of self-stigma and previous negative experiences with psychotherapy are associated with lower willingness to engage in online psychotherapy.

## Methods

### Study Design and Sample

This study is a cross-sectional, multicenter survey using a structured questionnaire to assess the attitudes and willingness of patients with mental disorders in China to engage in online psychotherapy. We collected data during a 2-month period from February through March 2025. Eight medical institutions across China were involved: 4 specialized psychiatric hospitals—Peking University Sixth Hospital in Beijing, the Second Affiliated Hospital of Xinxiang Medical University in Henan province, the Third People’s Hospital of Lincang in Yunnan province, and Ji’ao Brain Hospital of Siping in Jilin province—and 4 general hospitals with psychiatry departments—Zhongshan Hospital (Xiamen), Fudan University in Fujian province, First Hospital of Hebei Medical University in Hebei province, Shaanxi Nuclear Industry 215 Hospital in Shaanxi province, and Heping Hospital Affiliated to Changzhi Medical College in Shanxi province.

These institutions covered provinces spanning eastern (Beijing, Fujian, and Hebei), central (Henan and Shanxi), western (Yunnan), and northeastern (Jilin and Shaanxi) regions, including megacities (Beijing), medium-sized cities (Shijiazhuang), and peripheral cities (Lincang), ensuring representation of urban and rural populations and socioeconomic diversity. Attending physicians approached potential participants, and if a patient met the diagnostic criteria for a psychiatric disorder according to the *International Classification of Diseases*, *Tenth Revision*, they could be recommended to participate in the study. Specifically, the inclusion criteria were individuals who (1) were aged ≥10 years, (2) had a diagnosis of a mental disorder according to the International Classification of Diseases (tenth revision) criteria, (3) were able to read and complete the questionnaire, and (4) provided voluntary consent to participate in this survey. At each site, researchers distributed the structured questionnaire via quick response codes generated on the Wenjuanxing platform, which outpatients or inpatients scanned to complete the survey on their mobile phones.

On the basis of previous research [18], we assumed a 65% preference rate for online psychotherapy and calculated a minimum sample size of 350 participants (*Z*=1.96; *E*=0.05; *P*=.65) using standard proportion sampling methodology as follows:



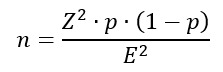



To account for invalid questionnaires (approximately 10%), we increased the sample size to 385 participants.

### Ethical Considerations

This study was approved by the Ethics Committee of Peking University Sixth Hospital. The approval number is 2025-1-9-1 (rapid review). Informed consent was obtained from all participants prior to enrollment, and participants were informed that their responses would be kept confidential. Parental or guardian consent was mandatory for all participants aged <18 years, in addition to the minor’s assent. To protect privacy, all data were anonymized, and no personally identifiable information was collected or stored. Data were stored on a secure, password-protected server accessible only to the research team. Participants received CNY ¥10 (US $1.40) as compensation for their time and participation.

### Measures

#### Demographics

Sex, age, educational attainment, occupational status, marital status, and socioeconomic status were collected in the survey. The exchange rate used for currency conversion during the study period was US $1=CNY ¥7.2.

#### Use Patterns of Internet-Enabled Devices

Participants indicated their primary internet access devices (eg, computer, tablet, and smartphone) as well as their frequency of internet use and their frequency of using video or voice call functions through multiple-choice or single-choice questions in the questionnaire.

#### Current Psychiatric Diagnoses and Treatment Experiences

Participants provided information regarding their diagnosis and treatment status by selecting the options that best matched their situation. The questionnaire covered several dimensions, including current diagnosis, pharmacotherapy (encompassing use, satisfaction, and disease improvement), and online psychotherapy (encompassing history of receiving treatment and satisfaction with it).

#### Willingness to Engage in Online Psychotherapy

Willingness refers to an individual’s conscious intent or readiness to engage in a specific future behavior, given the opportunity. It is a subjective probability judgment about one’s likelihood to act, positioned as a direct antecedent and key predictor of actual behavior [19]. In this study, “willingness to engage in online psychotherapy” was operationalized as participants’ self-reported likelihood of initiating or participating in this intervention in the future, assessed before any direct experience with it. We measured this construct using a digitized, single-item visual analogue scale, presented as an interactive horizontal slider ranging from 0 (“absolutely unwilling”) to 100 (“extremely willing”). A draggable indicator was initially placed at the midpoint (50). Participants were instructed to drag the indicator to the position that best reflected their current willingness. The web-based survey platform automatically recorded the corresponding numeric value (range 0-100) with 1 decimal place precision upon page submission.

#### Perceived Benefits and Barriers

Perceived benefits and barriers of online psychotherapy were assessed using 2 separate interactive drag-and-drop ranking tasks. One task listed 6 potential advantages, and the other listed 7 potential barriers to online psychotherapy. For each task, participants viewed all items in a randomized initial order. The instruction read: “please rank the following items in order of their importance to you, from the most important (1) to the least important (6 or 7), by dragging and dropping them into your preferred order.”

#### Preferences

We assessed preferences for various online psychotherapy modalities (eg, individual, group, or virtual reality therapy) by asking participants to rank them from most to least preferred. Participant preferences in relation to the per-session fee and the desired therapy frequency were captured via single-choice selection of the most preferred option for each item.

#### Stigma

We used the Self-Stigma of Seeking Help Scale (SSOSH) to assess help-seeking stigma and explore its association with online psychotherapy willingness [20]. The SSOSH scale is a 10-item self-report measure assessing the internalized stigma associated with seeking psychological help. Items (eg, “I would feel inadequate if I went to a therapist for psychological help”) are rated on a 5-point Likert scale ranging from 1 (strongly disagree) to 5 (strongly agree). The total score can range from 10 to 50, with higher scores indicating greater levels of self-stigma. The scale has demonstrated good reliability and validity in various populations [21]. Zuo and Ai [22] translated and revised the Chinese version of the SSOSH, with an internal consistency coefficient ranging from 0.86 to 0.90. In our study, the internal consistency coefficient (Cronbach α) for the SSOSH was 0.70, which is acceptable for a multi-item scale in this context. The questionnaire used in this study is provided in Multimedia Appendix 1.

### Statistical Analysis

Descriptive analyses included means with SDs for normally distributed continuous variables and counts with percentages for categorical variables. On the basis of the city information obtained from patients’ questionnaire responses, we classified the cities into 3 tiers according to urban hierarchy classification standards: large cities (urban resident population >1 million), medium-sized cities (urban resident population 500,000-1,000,000), and small cities (urban resident population <500,000) [23].

In the analysis of factors influencing online psychotherapy willingness, we used both univariate and correlation analyses.

Categorical variables were analyzed using the chi-square test. For continuous variables, normality was assessed using the Shapiro-Wilk test and visual inspection of quantile-quantile plots. Normally distributed data were analyzed using independent *t* tests (2 groups) or 1-way ANOVA (≥3 groups), with Fisher least significant difference post hoc tests applied if ANOVA was significant (*P*<.05). Nonnormal data were compared using Mann-Whitney *U* tests (2 groups) or Kruskal-Wallis tests (≥3 groups), followed by Dunn tests if overall differences existed (*P*<.05).

Spearman correlation analysis was used to examine relationships between continuous variables (age and SSOSH scores) and willingness to engage in online psychotherapy.

Multivariate linear regression analyses were used to identify independent factors associated with willingness to engage in online psychotherapy. Variables with statistical significance in the univariate analyses were entered into the regression model.

All analyses were performed using SPSS (version 26.0; IBM Corp). *P*<.05 was considered statistically significant based on 2-tailed *t* tests.

## Results

### Participant Characteristics

A total of 390 questionnaires were collected. Following a rigorous review process, 29 duplicate submissions (characterized by identical IP addresses and fully consistent questionnaire content) were excluded. Consequently, 361 valid questionnaires were retained for the final analysis.

In the total sample, females accounted for 65.1% (235/361), with a mean age of 32.1 (SD 11.9) years. Individuals with a primary or secondary education level constituted 40.4% (146/361), while married participants represented 39.9% (144/361). Those holding full-time or part-time employment accounted for 48.2% (174/361). In total, 51.8% (187/361) had an annual income below CNY ¥50,000 (US $6944.40). The participants’ addresses indicated residence in large, medium, and small cities at rates of 29.6% (107/361), 43.8% (158/361), and 26.6% (96/361), respectively. The smartphone use rate was 98.1% (354/361), and 78.1% (282/361) of participants used video platforms at least once a week. Among them, 65.4% (236/361) received outpatient psychiatric treatment, 62.3% (225/361) were diagnosed with anxiety or depression, and 69.0% (249/361) were on medication (details are provided in Table 1).

**Table 1:** Descriptive characteristic of the whole sample (N=361).

Variables		Values
**Sex, n (%)**		
	Female	235 (65.1)
	Male	114 (31.6)
	Other	1 (0.3)
	Prefer not to disclose	11 (3.0)
Age^a^ (years), mean (SD)		32.1 (11.9)
**Educational level, n (%)**		
	Primary school	24 (6.6)
	Junior high school	79 (21.9)
	Senior high school	43 (11.9)
	Higher vocational education	58 (16.1)
	Bachelor degree	127 (35.2)
	Master degree	26 (7.2)
	Doctoral degree	4 (1.1)
**Occupational status, n (%)**		
	Full time	133 (36.8)
	Part time	41 (11.4)
	Unemployed	51 (14.1)
	Retired	11 (3.0)
	Other	125 (34.6)
**Marital status, n (%)**		
	Married	144 (39.9)
	Unmarried, divorced, or widowed	217 (60.1)
**Annual income^b^** **(¥), n (%)**		
	≤10,000	107 (29.6)
	10,001-50,000	80 (22.2)
	50,001-100,000	36 (10.0)
	100,001-200,000	33 (9.1)
	200,001-500,000	11 (3.0)
	>500,000	2 (0.6)
	Prefer not to disclose	92 (25.5)
**City of residence, n (%)**		
	Large city	107 (29.6)
	Medium-sized city	158 (43.8)
	Small city	96 (26.6)
**Devices used, n (%)**		
	Smartphones	354 (98.1)
	Laptops	168 (46.5)
	Tablets	132 (36.6)
	Desktop computers	65 (18.0)
**Internet use frequency, n (%)**		
	Almost always	227 (62.9)
	Several times a day	95 (26.3)
	About once a day	12 (3.3)
	Several times a week	13 (3.6)
	Once a week	5 (1.4)
	Less than once a week	1 (0.3)
	Never	8 (2.2)
**Video platform use frequency, n (%)**		
	Almost always	78 (21.6)
	Several times a day	71 (19.7)
	About once a day	32 (8.9)
	Several times a week	78 (21.6)
	Once a week	23 (6.4)
	Less than once a week	56 (15.5)
	Never	23 (6.4)
**Current psychiatric diagnoses, n (%)**		
	Anxiety disorder	127 (35.2)
	Depressive disorder	98 (27.1)
	Bipolar disorder	43 (11.9)
	Sleep disorder	34 (9.4)
	Psychotic disorder	22 (6.1)
	Obsessive-compulsive disorder	21 (5.8)
	Substance dependence–related disorder	3 (0.8)
	Trauma-related disorder	2 (0.6)
	Other	11 (3.1)
**Illness duration (months), n (%)**		
	<6	96 (26.6)
	6-24	91 (25.2)
	>24	174 (48.2)
**Currently taking psychiatric medications, n (%)**		
	Currently taking	249 (69.0)
	Not taking	112 (31.0)
**Current treatment setting, n (%)**		
	Outpatient care	236 (65.4)
	Inpatient care	95 (26.3)
	Partial hospitalization	9 (2.5)
	Other	21 (5.8)
**Previous experience with online psychotherapy, n (%)**		
	Had experience	90 (24.9)
	No experience	271 (75.1)
Willingness to engage in online psychotherapy, mean (SD)		70 (28.56)
**Acceptable cost per session for online psychotherapy (¥), n (%)**		
	≤200	285 (78.9)
	201-400	49 (13.6)
	401-600	13 (3.6)
	>600	14 (3.9)
**Preferred number of sessions for online psychotherapy, n (%)**		
	1-5	212 (58.7)
	6-10	100 (27.7)
	11-15	32 (8.9)
	>15	17 (4.7)
Self-Stigma of Seeking Help Scale scores, mean (SD)		25.04 (5.56)

^a^n=354 participants.

^b^Exchange rate during the study period: US $1=CNY ¥7.2.

### Online Psychotherapy Willingness, Perceived Benefits, and Barriers

**Figure 1:**
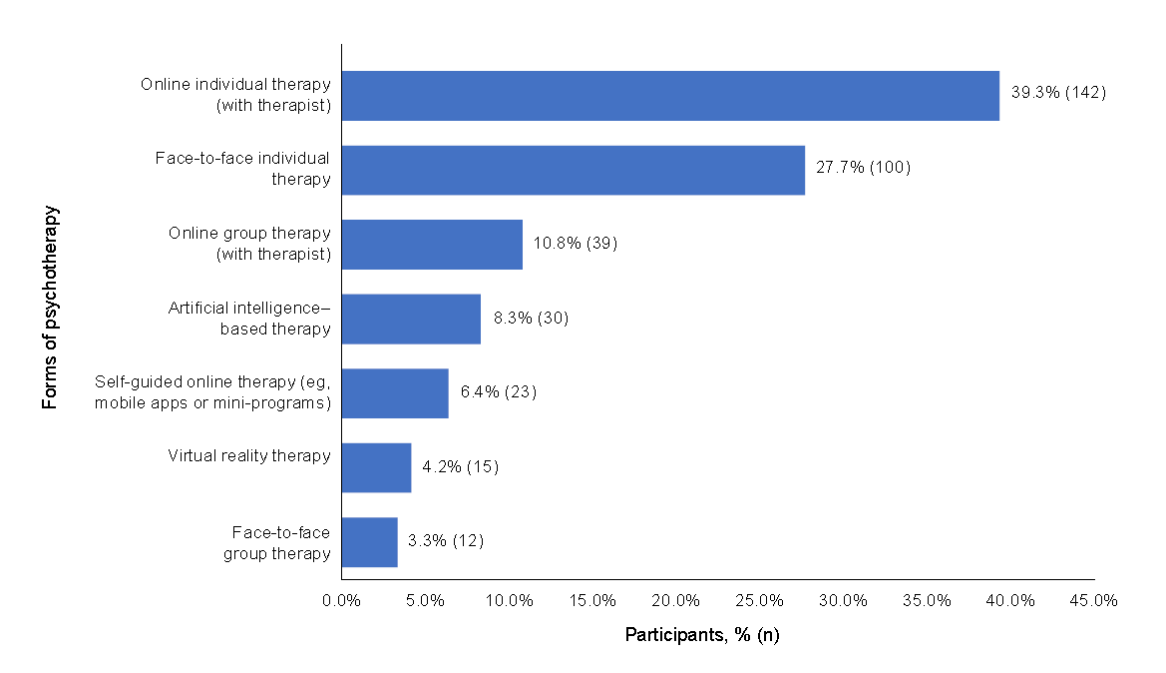
Ranking of preferences for different forms of psychotherapy.

In our study, the mean willingness score for engaging in online psychotherapy was 70 (SD 28.56). In total, 86.4% (312/361) of participants preferred short-term therapy (1-10 sessions), while 92.5% (334/361) expected the cost per session to remain less than ¥400 (details are provided in Table 1). Regarding the formats of psychotherapy, participants most preferred therapist-guided online individual therapy (142/361, 39.3%), which was higher than face-to-face individual therapy, whereas technology-assisted formats (artificial intelligence, virtual reality, and self-guided) showed modest acceptance (68/361,18.8%). Face-to-face group therapy ranked lowest (12/361, 3.3%; Figure 1).

**Figure 2:**
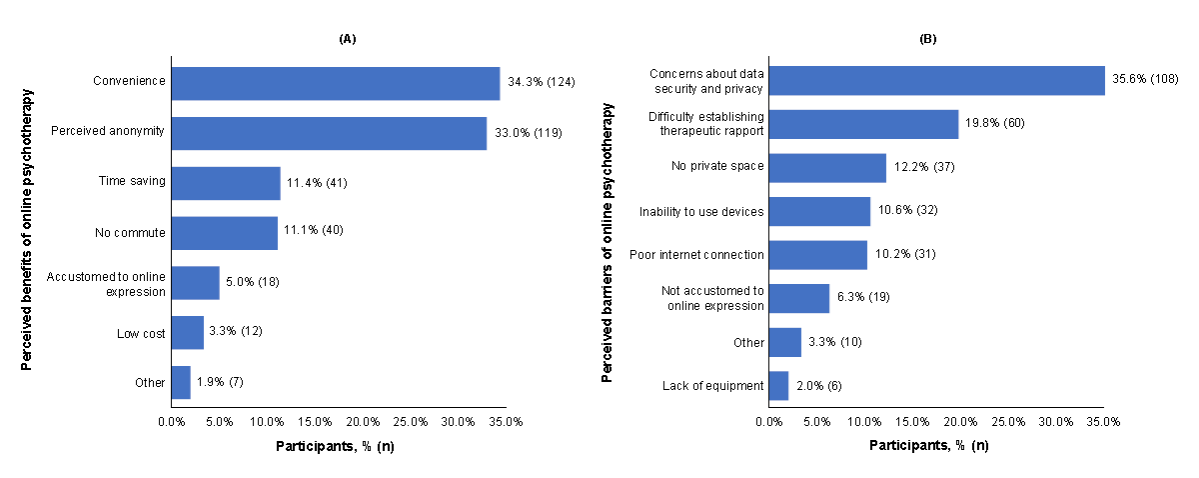
Perceived benefits and barriers of online psychotherapy, ranked from 1 (most important) to 6 (least important). (A) What are the benefits of online psychotherapy (n=361)? and (B) What stops you from trying online psychotherapy (n=303)?

Figure 2 shows participants’ perceived benefits and barriers of online psychotherapy. We found that convenience (124/361, 34.3%) and perceived anonymity (“no one will know about the illness”; 119/361, 33.0%) were the 2 most commonly perceived benefits of online psychotherapy. Concerns about data security and privacy (108/303, 35.6%) emerged as the most significant barrier, followed by difficulty in establishing therapeutic rapport (60/303, 19.8%).

### Factors Associated With Online Psychotherapy Willingness

Univariate analyses revealed several factors significantly associated with willingness to engage in online psychotherapy, as shown in Table 2.

Some of the key factors from Table 2 are geographic variation, treatment status, and previous experience. For geographic variation, participants from small cities demonstrated significantly higher willingness than those from medium or large cities. For treatment status, individuals currently using psychiatric medications showed a higher willingness than those not using medications. Those reporting being “very satisfied” with pharmacotherapy were more willing than those in other satisfaction categories. Individuals with previous online psychotherapy experience exhibited greater willingness than those without such experience. Participants who were satisfied with past online psychotherapy were more willing than those who were neutral or dissatisfied.

Spearman rank correlation revealed a weak but statistically significant positive association (ρ=0.17; *P*=.001) between age and online psychotherapy willingness. A statistically significant negative correlation of comparable magnitude was observed (ρ=−0.17; *P*=.002) between total SSOSH score and online psychotherapy willingness. We found that participants preferring online psychotherapy had significantly higher SSOSH scores than those preferring face-to-face sessions (mean 25.53, SD 5.31 vs mean 23.96, SD 5.96; *P*=.01). The regression model incorporated all statistically significant variables as independent variables (including age, city size, use of psychiatric medications, satisfaction with pharmacotherapy, previous online psychotherapy experience, satisfaction with previous online psychotherapy, and SSOSH scores), with willingness to engage in online psychotherapy as the dependent variable. The regression analysis revealed that age, total SSOSH score, satisfaction with current psychiatric medications, and satisfaction with previous online psychotherapy significantly influenced patients’ willingness to seek online psychotherapy (*P*<.05; details are provided in Table 3). The model demonstrated good explanatory power, with an *R*^2^ =0.518 and adjusted *R*^2^=0.475. Additionally, diagnostic checks revealed that the residuals were normally distributed (Shapiro-Wilk *P*=.18), exhibited homoscedasticity, and showed no issues with multicollinearity (variance inflation factor<2.5).

**Table 2:** Univariate analysis of factors associated with willingness to seek online psychotherapy (N=361).

Variables		Participants, n (%)	Willingness score, mean (SD)	*F* value (*df*)		*t* value (*df*)		*P* value^a^		η^2^		Cohen *d*	
**Sex^c^**					0.577 (2)		—^b^		.56		0.003		—
	Female	235 (65.1)	70.43 (28.63)										
	Male	114 (31.6)	70.03 (29.01)										
**Age^d^** **(years)**					2.628 (5)		—		*.02*		0.036		—
	<20	42 (11.9)	64.26 (28.77)										
	20-29	143 (40.4)	68.68 (26.99)										
	30-39	90 (25.4)	69.34 (26.37)										
	40-49	45 (12.7)	76.51 (31.55)										
	50-59	26 (7.3)	83.27 (28.52)										
	≥60	8 (2.3)	53.00 (44.44)										
**City size^e^**					2.872 (2)		—		.06		0.016		—
	Large	107 (29.6)	67.22 (29.45)										
	Medium	158 (43.8)	68.28 (26.12)										
	Small	96 (26.6)	75.91 (30.79)										
**Occupation**					0.645 (4)		—		.63		0.007		—
	Full time	133 (36.8)	70.22 (27.94)										
	Part time	41 (11.4)	73.12 (26.68)										
	Unemployed	51 (14.1)	73.84 (25.85)										
	Retired	11 (3.0)	65.73 (37.31)										
	Others	125 (34.6)	67.54 (30.15)										
**Education level**					1.768 (5)		—		.12		0.024		—
	Primary school	24 (6.6)	69.50 (34.58)										
	Junior high school	79 (21.9)	73.65 (30.10)										
	Senior high school	43 (11.9)	72.58 (30.42)										
	Higher vocational education	58 (16.1)	63.24 (30.60)										
	Bachelor degree	127 (35.2)	67.85 (26.07)										
	Graduate degree (master or doctoral)	30 (8.3)	79.23 (19.04)										
**Annual income^f^** **(¥)**					1.427 (5)		—		.21		0.020		—
	<10,000	107 (29.6)	68.36 (28.94)										
	10,001-50,000	80 (22.2)	75.28 (27.71)										
	50,001-100,000	36 (10.0)	69.39 (28.33)										
	100,001-200,000	33 (9.1)	73.12 (27.37)										
	>200,000	13 (3.6)	78.23 (19.41)										
	Prefer not to disclose	92 (25.5)	65.27 (30.00)										
**Illness duration (months)**					0.827 (2)		—		.44		0.005		—
	<6	96 (26.6)	67.14 (27.34)										
	6-24	91 (25.2)	69.62 (26.30)										
	>24	174 (48.2)	71.78 (30.32)										
**Currently taking psychiatric medication**					—		3.082 (359)		*.002*		—		0.351
	Currently taking	249 (69.0)	73.07 (28.01)										
	Not taking	112 (31.0)	63.17 (28.72)										
**Satisfaction with psychiatric medication^g^**					11.853 (4)		—		*<.001*		0.155		—
	Very satisfied	77 (30.9)	89.27 (18.64)										
	Somewhat satisfied	115 (46.2)	66.95 (27.39)										
	Neutral	51 (20.5)	64.45 (29.56)										
	Somewhat dissatisfied	5 (2.0)	62.80 (42.69)										
	Very dissatisfied	1 (0.4)	20.00^h^										
**Previous experience with online psychotherapy**					—		3.478 (359)		*.001*		—		0.423
	Experienced	90 (24.9)	78.93 (25.09)										
	Not experienced	271 (75.1)	67.03 (29.07)										
**Satisfaction with previous online psychotherapy^i^**					7.633 (4)		—		*<.001*		0.264		—
	Very satisfied	13 (14.4)	88.38 (27.40)										
	Somewhat satisfied	35 (38.9)	91.20 (14.76)										
	Neutral	25 (27.8)	67.60 (22.90)										
	Somewhat dissatisfied	10 (11.1)	57.00 (27.62)										
	Very dissatisfied	7 (7.8)	71.86 (29.84)										

^a^Not applicable.

^b^Italicized values indicate variables with statistically significant differences (*P*<.05).

^c^n=349; 12 participants did not disclose their gender or entered “other.”

^d^n=354 participants; post hoc comparisons (Fisher least significant differences test or Dunn test) showed higher willingness scores among participants aged 50-59 years than those aged <20, 20-29, 30-39, and ≥60 years, and among those aged 40-49 years than those aged <20 and ≥60 years.

^e^Post hoc comparisons showed higher willingness scores among participants from small cities than those from large and medium cities.

^f^Exchange rate during the study period: US $1=CNY ¥7.2.

^g^n=249.

^h^n=1, SD not applicable.

^i^n=90, post hoc comparisons showed higher willingness scores among participants who were somewhat satisfied than those who were neutral, somewhat dissatisfied, or very dissatisfied, and among those who were very satisfied than those who were neutral and very dissatisfied.

**Table 3:** Multiple linear regression predicting willingness to seek online psychotherapy.

Variables	B (SE)	β	*t* test (*df*)	*P* value
Age	0.465 (0.176)	0.276	2.642 (67)	.01
Total Self-Stigma of Seeking Help Scale score	−0.815 (0.407)	−0.205	−2.004 (67)	.049
Satisfaction with psychiatric medication	11.193 (3.008)	0.369	3.721 (67)	<.001
Satisfaction with previous online psychotherapy	6.250 (1.960)	0.303	3.189 (67)	.002

## Discussion

This study is a nationwide, multicenter, cross-sectional survey, and participants come from both specialized psychiatric hospitals and general hospitals with psychiatric departments in China. Our findings indicate a moderate level of willingness to engage in online psychotherapy, with a preference for short-term, affordable, therapist-guided individual formats. Convenience and perceived anonymity were key perceived benefits, while data security concerns and challenges in establishing therapeutic rapport were major barriers. Importantly, age, satisfaction with pharmacotherapy, previous positive experiences with online psychotherapy, and levels of self-stigma (SSOSH scores) independently predicted patients’ willingness, highlighting critical leverage points for expanding access to digital mental health services.

Our study reveals that Chinese patients with mental disorders exhibit moderate-to-high willingness to engage in online psychotherapy, indicating promising prospects for its application in China. Previous studies have found that fewer than 10% of individuals with mental disorders seek professional help, which is associated with China’s vast territory, uneven distribution of mental health resources, and concentration of psychotherapy services primarily in major cities [24,25]. In response, the National Health Commission has launched a 3-year initiative (2025-2027) to strengthen pediatric and mental health services (National Health Commission of the People’s Republic of China, 2025). Online psychotherapy—with its advantages in convenience, privacy, and cost and time efficiency—aligns well with patients’ treatment needs. Expanding such services may provide an important avenue for improving the accessibility of mental health care delivery in China.

Regarding factors influencing willingness to engage in online psychotherapy, univariate analysis revealed that (1) age was positively correlated with willingness, (2) participants from small cities demonstrated significantly higher willingness than those from medium or large cities, (3) individuals satisfied with their current psychopharmacological treatment or with previous online psychotherapy experience exhibited greater willingness, and (4) the total SSOSH score showed a negative association with online psychotherapy willingness. Multivariate analysis revealed that age, satisfaction with current medication, and satisfaction with previous psychotherapy exerted positive effects on willingness, while the total SSOSH score showed a negative association. Previous studies indicate age-related differences in online mental health resource use: tech-savvy younger individuals show greater adoption, while middle-aged users prefer traditional authoritative sources. Older adults’ use is often limited by digital barriers and physical or cognitive constraints [14,26]. However, in our study, we found that middle-aged patients demonstrated a higher willingness for online psychotherapy than younger patients. One possible explanation for this age-related difference is that middle-aged individuals often face compounded work and family responsibilities, which may heighten their sensitivity to the flexibility and efficiency offered by online modalities. Although these underlying factors (such as perceived time pressure or convenience needs) were not directly assessed in our study, they represent a plausible interpretation grounded in the existing literature on midlife developmental tasks [27,28]. Future studies should incorporate direct measurements of these psychological and practical barriers to empirically test the mediating mechanisms driving the relationship between age and willingness to use online psychotherapy. Mental health resources in China are unevenly distributed, with in-person psychotherapy services being more accessible in large cities but significantly scarcer in smaller urban areas [25]. Online psychotherapy effectively breaks down geographic barriers, enabling patients from smaller cities to access higher-quality services typically concentrated in major metropolitan areas. This may explain why residents of smaller cities exhibit greater willingness to adopt online psychotherapy than those in medium-sized and large cities. Participants satisfied with pharmacotherapy and previous psychotherapy exhibited greater willingness to pursue online psychotherapy. This suggests that effective medication treatment may serve as a gateway to psychological interventions, as symptom improvement motivates patients to seek further quality-of-life enhancements. For clinically indicated patients with low treatment motivation, successful pharmacotherapy demonstrates dual benefits: not only relieving symptoms but also facilitating potential long-term engagement in psychotherapy. Finally, stigma remains a significant barrier to treatment seeking among patients, with higher stigma levels correlating with lower treatment willingness [29]. However, online psychotherapy may present a preferable alternative for highly stigmatized individuals due to its enhanced privacy and convenience. Previous studies have shown that patients with higher stigma scores tend to prefer online psychotherapy over face-to-face therapy [30]. Our study yielded similar findings: stigma scores negatively correlated with willingness to pursue psychotherapy overall; however, among treatment format preferences, patients who preferred online psychotherapy exhibited higher stigma scores than those favoring face-to-face therapy.

Regarding the formats and settings of online psychotherapy, we found that participants preferred therapist-guided, individual online psychotherapy, particularly short-term interventions (1-10 sessions) priced <¥400 per session. Therapist-guided psychotherapy remained participants’ preferred choice, suggesting that even when integrating technologies such as virtual reality, artificial intelligence, or self-help applications, maintaining a therapeutic role for clinicians remains essential. To enhance accessibility and engagement, future online psychotherapy services could adopt modular, short-term treatment plans with flexible themes and phases, allowing patients greater autonomy in tailoring their therapeutic journey. These adaptations could make online psychotherapy more accessible and sustainable.

The primary obstacles preventing patients from seeking online psychotherapy were concerns about data security and privacy, followed by difficulties in establishing therapeutic relationships. Other obstacles include the absence of a private space suitable for online psychotherapy, difficulty using devices, or poor internet connection, illustrated in Figure 2. These findings are largely consistent with previous research [25,31,32]. To address these barriers, it is essential to enhance data security and privacy protection to mitigate the risk of information breaches. It will be helpful to provide specialized training for therapists to develop therapeutic relationships in virtual environments, thereby reducing difficulties in establishing rapport. Additional improvement measures include offering pretherapy guidance on the use of digital devices to minimize technical barriers and their negative impact. Future efforts should focus on further refining the standards for online psychotherapy to comprehensively enhance its accessibility and effectiveness.

Our study has some limitations. First, patients with anxiety and depression accounted for 62.3% (225/361) of the sample. Although other disorders were less represented, the study still covered common mental health conditions such as bipolar disorder, sleep disorders, obsessive-compulsive disorder, trauma-related disorders, substance dependence, and psychotic disorders. Future studies should investigate specific patient populations to provide tailored references for implementing online psychotherapy for patients with different disorders. Second, our sample was recruited from hospital settings, which ensured that all participants had clinically confirmed mental disorders; however, it may have introduced selection bias toward higher treatment-seeking motivation and consequently elevated willingness to engage in online psychotherapy. Consequently, the overall willingness reported in this study may be overestimated compared to community-dwelling or untreated populations, which limits the generalizability of our findings to broader, non–treatment-seeking groups. Future studies should include community-based samples to improve external validity and allow for more comprehensive comparisons across different subgroups. Third, while we included sociodemographic and treatment variables in the regression analysis, factors such as illness severity may also shape willingness. Future studies need to consider other potential influencing factors.

In conclusion, this study provides novel evidence from a multicenter clinical sample of Chinese patients with mental disorders, revealing not only a relatively high willingness to engage in online psychotherapy but also distinct preferences for specific treatment formats. Furthermore, we identified self-stigma (measured by the SSOSH) as an independent negative predictor of willingness. These findings have several practical implications. First, online psychotherapy services should be tailored to patient preferences by offering flexible modality options. Second, targeted interventions aimed at reducing self-stigma could be integrated into digital platforms to lower psychological barriers, particularly for patients in smaller cities where access to traditional mental health services is limited but there is a high willingness to engage in online psychotherapy. Finally, policymakers and health care administrators should consider investing in infrastructure and training programs that support the scalable implementation of online psychotherapy, thereby strengthening the reach and resilience of mental health care delivery in China.

## Appendix

Multimedia Appendix 1 Self-Stigma of Seeking Help (SSOSH) scale.

## Abbreviations

SSOSH: Self-Stigma of Seeking Help Scale
