# Supplementary material for: Willingness of Patients With Mental Disorders to Engage in Online Psychotherapy: Multicenter Cross-Sectional Survey
Source: JMIR Form Res. 2026 Apr 7;10:e83299. doi: 10.2196/83299 (PMC13100576; doi:10.2196/83299)
Supplement: Multimedia Appendix 1 [file formative_v10i1e83299_app1.docx]

Self-Stigma of Seeking Help (SSOSH)

The Self-Stigma of Seeking Help (SSOSH) is a 10-item scale to measure the construct of self-stigma in the context of engaging in therapy.

Items:

I. I would feel inadequate if I went to a therapist for psychological help.

2. My self-confidence would NOT be threatened if I sought professional help.*

3. Seeking psychological help would make me feel less intelligent.

4. My self-esteem would increase if I talked to a therapist.*

5. My view of myself would not change just because I made the choice to see a therapist.*

6. It would make me feel inferior to ask a therapist for help.

7. I would feel okay about myself if I made the choice to seek professional help.*

8. If I went to a therapist, I would be less satisfied with myself.

9. My self-confidence would remain the same if I sought professional help for a problem I could not solve.*

10. I would feel worse about myself if I could not solve my own problems.

Response Options:

5-point Likert scale

Strongly disagree - 1

Disagree - 2

Agree and disagree equally - 3

Agree - 4

Strongly agree - 5

*Items are reverse-scored

Scoring Procedures

The mean of all items is calculated for the total score. Higher scores indicated a greater concern that seeking help from a psychologist or other mental health professional would negatively affect one’s self regard, satisfaction with oneself, self-confidence, and overall worth as a person.

Original Citation

Vogel, D. L., Wade, N. G., & Haake, S. (2006). Measuring the self-stigma associated with seeking psychological help. Journal of Counseling Psychology, 53(3), 325-337. https://doi.org/10.1037/0022-0167.53.3.325
